# Late recurrence of malignant melanoma presenting with hemoptysis

**DOI:** 10.4103/0970-2113.68320

**Published:** 2010

**Authors:** Karanam Gowrinath, Vasudevan Geetha

**Affiliations:** *Department of Pulmonary Medicine and Pathology, Kasturba Medical College, Manipal, Karnataka State, India*

**Keywords:** Hemoptysis, malignant melanoma, metastatic malignant melanoma

## Abstract

After a disease-free period of 10 years, a surgically treated case of cutaneous malignant melanoma is usually not followed up further and there is a tendency to assume that the disease is cured. Late recurrence (after 10 years) of cutaneous malignant melanoma, though infrequent, has been documented well in Western countries. In our country, the malignant melanoma is still considered uncommon and there is no data regarding its late recurrence. We report a case of pulmonary malignant melanoma as a late metastatic manifestation of primary plantar malignant melanoma in a 61-year-old man who presented with hemoptysis; metastatic malignant melanoma of the lung occurred 12 years after resection of primary malignant melanoma of sole of the right foot.

## INTRODUCTION

Malignant melanoma is common among White skinned individuals who have approximately 10 times greater risk of developing cutaneous melanoma than Black, Asian or Hispanic population.[1] But the incidence of plantar malignant melanoma is equal in both the White and Black population.[2] In India, malignant melanoma is uncommon and its incidence was less than 0.5%.[3] Most cases of primary cutaneous malignant melanoma are cured by surgical excision but 30% of them may develop metastatic lesions later, often in the lung.[4] Recurrence of malignant melanoma after 10 years of apparent cure is uncommon and its incidence is 0.65%-6.7%.[5,6] We report late recurrence of malignant melanoma in a surgically treated case of primary plantar malignant melanoma.

## CASE REPORT

**Figure 1:**
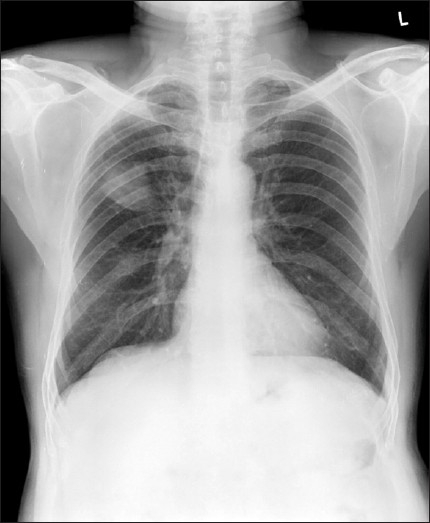
Chest radiograph showing a solitary pulmonary mass lesion in the right upper lobe

**Figure 2:**
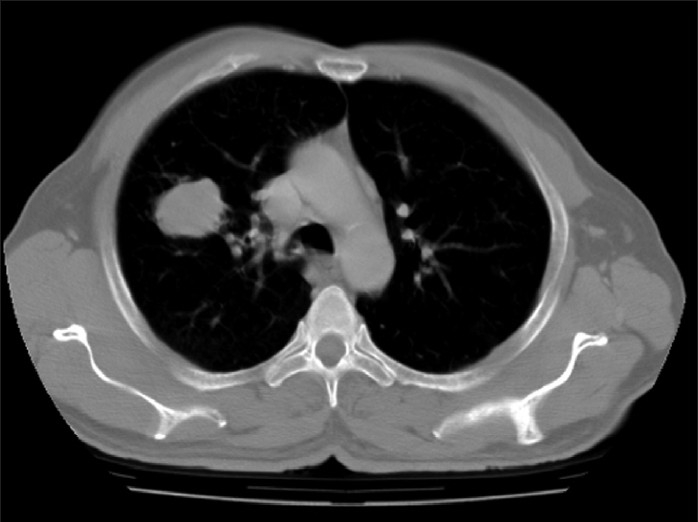
CT scan of chest showing the mass lesion in the anterior segment of right upper lobe with smooth lobulated margins

**Figure 3:**
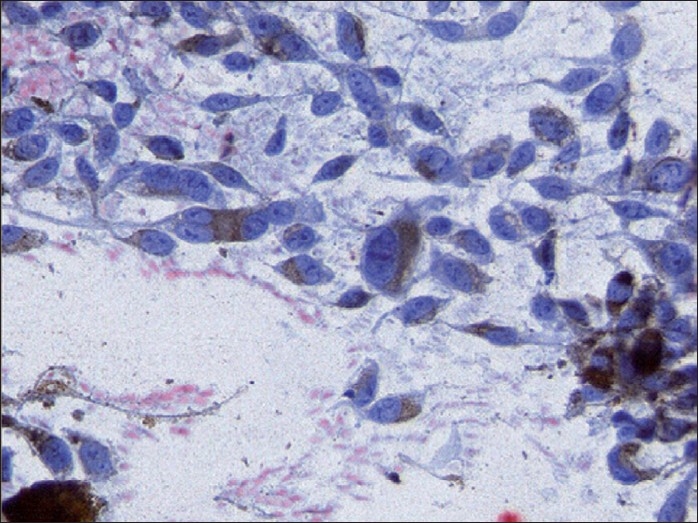
Spindle shaped neoplastic melanocytes with large nuclei, some with nucleoli and abundant cytoplasmic melanin (PAP × 400)

A 61-year-old man presented with hemoptysis of three months duration. Hemoptysis was minor and intermittent. There was no history of fever. Appetite was normal. Medical history was significant for asthma for the past 15 years and patient has been using inhaled bronchodilators irregularly. He underwent partial resection of right foot 12 years ago for malignant melanoma of sole. Before presenting to us, the patient received antituberculous therapy consisting of isoniazid, rifampicin and ethambutol for two months at local place without relief. The basis for starting antituberculous treatment was abnormal radiographic finding alone. An ex-smoker, he is a farmer by occupation. Physical examination showed digital clubbing and the partially resected right foot. There was no significant lymphadenopathy. Clinical examination of all the systems including the eyes and skin were normal. Blood investigations showed a hemoglobin of 15.1gms%, total leukocyte count of 7,200/mm^3^ with a differential of 70% neutrophils and 22% lymphocytes. Erythrocyte sedimentation rate was 10mm in the first hour. Blood chemistry was normal. Chest radiograph [Figure 1] showed a solitary pulmonary mass within the right upper lobe. Electrocardiogram was normal. HIV serology was negative. Flexible bronchoscopy findings were normal. Computed tomographic (CT) scan of chest [Figure 2] showed a mass lesion (2.6 × 3.7 × 4.0 cms) in the anterior segment of the right upper lobe with smooth lobulated margins. Ultrasound and CT scan study of abdomen were normal. Cytological examination of the needle aspirate of pulmonary lesion under CT guidance showed malignant cells with brown melanin pigment [Figure 3]. Surgical treatment was planned and the patient was referred to the cardiothoracic surgeon. But preoperative surgical biopsy was not possible as patient did not come for review after being discharged at his request and was lost to follow-up.

## DISCUSSION

Malignant melanoma is an aggressive neoplasm with unpredictable metastatic spread. Pulmonary metastatic melanomas are often asymptomatic, multiple and detected only through imaging techniques.[7] Most of the recurrences of malignant melanoma occur within 10 years after surgical treatment of primary neoplasm. In a large series involving more than 7000 cases of malignant melanoma, late recurrence occurred in 2.4% cases, 48.2% of them between 10 to 12 years with regional lymph nodes being the most common site of metastases followed by the lung.[8] Recurrence of primary malignant melanoma after 15 years of successful surgical treatment was reported in 2% of cases.[9] In our case, partial resection of right sole was done for primary malignant melanoma twelve years ago and he remained asymptomatic until hemoptysis occurred as a manifestation of metastatic melanoma. Since the patient had 12 years of disease-free period after surgical resection of plantar malignant melanoma, the local doctor suspected pulmonary tuberculosis as the cause of hemoptysis and the chest radiographic opacity.

Malignant melanoma within airways may present with hemoptysis.[10,11] In our case, the tumor is situated in the anterior segment of right upper lobe and hemoptysis was minor and intermittent. The initial radiologic presentation of metastatic malignant melanoma is more often as multiple nodules than as a solitary pulmonary nodule which was reported in 10% of cases.[12] Pulmonary metastasis as the sole clinical manifestation was documented in 7-9% cases of cutaneous malignant melanoma.[13]

In our case, both the clinical and imaging studies did not show metastatic lesions in other organs. The prognosis in case of distant metastases due to primary malignant melanoma is usually poor and without surgery, median survival with lung involvement was only 7.3 months.[14] A solitary pulmonary nodule due to metastatic malignant melanoma may respond well after complete resection of the nodule followed by chemotherapy as recommended before.[15] To our knowledge, this is the first Indian report of late recurrence of a malignant melanoma as a solitary pulmonary mass after successful resection of primary plantar melanoma. In conclusion, after surgical treatment of cutaneous malignant melanoma, 10 years of disease-free period does not represent cure and patient may need further life-long follow-up.
